# Cutaneous Squamous Cell Carcinoma in Epidermolysis Bullosa: A Review of Pathogenesis, Diagnosis and Management

**DOI:** 10.3390/cancers17193211

**Published:** 2025-10-01

**Authors:** Abarajithan Chandrasekaran, Justin C. Moser

**Affiliations:** 1 University of Arizona College of Medicine, Phoenix, AZ 85004, USA; 2 HonorHealth Research Institute, Scottsdale, AZ 85258, USA; 3 School of Medicine and Advanced Medical Engineering, Arizona State University, Tempe, AZ 85258, USA

**Keywords:** Recessive dystrophic epidermolysis bullosa, junctional epidermolysis bullosa, cutaneous squamous cell carcinoma, immunotherapy, anti-EGFR therapy

## Abstract

Epidermolysis bullosa (EB) is a group of debilitating, genetic skin disorders characterized by excessive skin fragility, blistering, and ulcerations that cause a cyclical wound healing process. EB presents itself in various subtypes, such as EB simplex (EBS), junctional EB (JEB), dystrophic (DEB), and Kindler Syndrome (KS), which all differ in their genetic cause, severity, and harbor different causes of mortality. Of these variants, JEB and DEB are the most severe, with EBS being the mildest form of the disease and KS presenting in extremely rare cases. The JEB variant tends to cause mortality early on in children less than two years of age due to failure to thrive, sepsis from wound infections, and airway obstruction. In the recessive form of DEB (RDEB), cutaneous squamous cell carcinoma (cSCC) is the major cause of death in patients, with one study reporting a mere 4-year survival after the first EB-cSCC diagnosis. Cutaneous SCCs in the setting of RDEB are particularly concerning because they are often more aggressive and show greater metastatic potential, as compared to ultraviolet-induced SCCs. This review aims to explore the pathophysiology of these EB variants as well as their implications for developing cSCCs. It will also discuss elements of the clinical presentation of such lesions in EB patients and the challenges associated with making a definitive diagnosis. Additionally, we will illuminate various diagnostic techniques, current and future management and treatment strategies for both cSCC and EB, and the importance of early screening and education for patients with EB to maximize patient lifespan and quality of life.

## Simple Summary

Epidermolysis bullosa (EB) and its subtypes represent debilitating, genetic skin conditions characterized by fragility, blistering, and wounds. Its pathology causes patients to be at an increased susceptibility for developing cutaneous squamous cell carcinoma (cSCC). cSCC in EB has proven to dramatically decrease the life expectancy of such patients since they are often undiagnosed and more susceptible to metastasis. This review aims to discuss the various subtypes of EB through their pathogenesis and clinical presentation, as well as that of cSCC in normal skin and in EB. We also discuss current methods of diagnosis and treatment, including surgical excision, immunotherapy, and anti-epidermal growth factor receptor (anti-EGFR) therapy. The review concludes with a discussion on future directions and additional research needed to decrease mortality in EB-associated cSCC.

## 1. Introduction

Epidermolysis bullosa (EB) is a diverse group of rare skin disorders characterized by specific mutations in the genes responsible for maintaining the structural integrity of the dermal-epidermal basement membrane, including *KRT5*, *KRT14*, *LAMA3*, *LAMB3*, *LAMC2*, *COL17A1*, *COL7A1*, and *FERMT1*. These mutations result in skin fragility, erosions, blister formation upon minor mechanical trauma, and are responsible for the pathogenesis of the various forms of EB. Such qualities of EB predispose patients to repeated cycles of cutaneous destruction and wound healing, thus increasing the likelihood of developing cutaneous squamous cell carcinoma (cSCC) [1]. Subtypes of EB vary in pathophysiology and thus severity of disease. The heterogeneous nature of EB highlights the fact that, currently, pathologic mutations in at least 20 unique genes can result in disease [1]. Despite this, inherited EB has been classified broadly into 4 main subtypes that reflect degrees of blistering: EB simplex (EBS), dystrophic EB (DEB), junctional EB (JEB), and Kindler Syndrome (KS) [2].

## 2. Subtypes of Epidermolysis Bullosa

### 2.1. EB Simplex

EBS accounts for approximately 70% of all EB cases. It is typically inherited in an autosomal dominant manner, although autosomal recessive inheritance may also occur [2]. The overwhelming majority of EBS cases arise from mutations in genes that control keratin production. Specifically, these cases originate from mutations in the *KRT5* and *KRT14* genes, both of which encode keratin proteins 5 and 14 [3]. Production of inadequate keratin can result in keratin aggregates that may play a role in endoplasmic reticulum (ER) stress by triggering phosphorylation by mitogen-activated protein kinases (MAPKs). This can then cause downstream effects, including poor proteasome degradation, abnormal cell migration, impaired adherence of keratinocytes, and more. Furthermore, the release of danger-associated molecular pattern (DAMP) molecules from the ER can trigger systemic chronic inflammation, as is seen in EBS [4]. Additional genetic causes of EBS can result in damaged hemidesmosomes and thus impaired cytoskeletal integrity in basal keratinocytes. Such mutations include changes in the plectin (PLEC), dystonin (DST), and exophilin 5 (EXPH5) proteins [2].

### 2.2. Junctional EB

Although JEB is significantly less common than EBS, accounting for about 5% of cases, its disease process is far worse because it presents with body-wide mucocutaneous blisters and erosions from birth. These lesions perpetuate losses in protein and fluids integral to protecting against infection. Accumulation of granulation tissue on the face, nail folds, and buttocks predisposes these patients to extreme pain, developmental slowing, anemia, and respiratory complications that lead to death in early infancy [5]. JEB is inherited in an autosomal recessive manner and can have more intermediate and severe forms. The generalized intermediate form involves mutations in the *LAMA3*, *LAMB3*, and *LAMC2* genes. These genes encode the a3, b3, and y2 chains of laminin-332 [6], a protein essential for the integrity of the dermal-epidermal basement membrane. In JEB, loss of this protein can subsequently lead to detachment of the epidermis from the dermis and thus predispose the skin to fragility and blistering due to mechanical stress [7]. Because laminin 332 is present in extracutaneous areas such as the brain, eyes, lungs, GI tract, and more, mutations in the protein can have consequences in other parts of the body, including pneumonia, dyspnea, tooth enamel defects, and pathologic granulation tissue involvement of the cornea, upper respiratory tract, and urinary tract [2,7].

The generalized severe form of the disease is due to a mutation in the *COL17A1* gene, which produces collagen 17. Collagen 17 is a crucial structural component of hemidesmosomes. This protein is primarily expressed by basal keratinocytes, allowing them to easily adhere to the underlying basement membrane. Lack of this protein ultimately results in generalized regions of skin blistering and mucous membranes, which can then lead to severe scarring [1]. Because JEB mutations result in the disruption of proteins involved in the structural maintenance of the lamina lucida, the layer of the basement membrane responsible for attachment of the epidermis to the dermis [6], consistent injury to this layer can increase the risk of developing cSCCs [8].

### 2.3. Dystrophic EB

DEB represents approximately 25% to 50% of cases and can be inherited in either an autosomal dominant or autosomal recessive pattern [9]. However, autosomal recessive genotypes are seen to produce more severe phenotypes. In the recessive form of DEB (RDEB), cutaneous squamous cell carcinoma (cSCC) is the major cause of death in patients, with one study reporting a mere 4-year survival after the first EB-cSCC diagnosis [9,10]. DEB can arise from mutations in the *COL7A1* gene that produces collagen 7. Normally, these proteins are responsible for anchoring the lower part of the dermal-epidermal basement membrane to the upper portion of the dermis underneath. In DEB, mutations to collagen 7 can cause deeper detachment of these skin layers, causing increased skin fragility and easy blistering [11]. The destruction of the sublamina densa as compared to the lamina lucida in JEB indicates that DEB may have more severe complications regarding scarring and fibrosis, and as a result, is the subtype that is typically associated with the highest risk of developing deeper and potentially metastatic cSCCs [9,12,13].

### 2.4. Kindler Syndrome

Kindler syndrome (KS) is the rarest variant of EB that is inherited in an autosomal recessive manner, with only about 400 cases reported globally [2]. Mutations in the *FERMT1* gene can result in defective copies of the kindlin-1 protein. Kindlin-1 normally localizes to keratinocytes in the basal skin layer at the dermal-epidermal junction and plays a role in activating integrin proteins [14]. Defects in kindlin-1 can thus result in compromised keratinocyte adhesion, proliferation, movement, and polarization. As a result, patients with KS will present with significant blistering of the skin, cutaneous atrophy, poikiloderma, and more. Most unique to KS is, however, elevated photosensitivity, causing almost immediate burning and erythema after sun exposure [15]. Photosensitivity in patients tends to improve with advanced age. Long-term complications from KS also include mild to aggressive cSCCs [16].

### 2.5. Acquired EB

Epidermolysis bullosa acquisita (EBA) is an autoimmune manifestation of EB that is characterized by autoantibodies to type VII collagen. This form of collagen is a major component of anchoring fibrils within the dermal-epidermal junction [11]. Damage to these anchoring fibrils subsequently results in cutaneous blistering via separation of the dermis and epidermis. EBA can be further classified into an inflammatory and non-inflammatory subtype. The inflammatory version of this disease closely mimics other autoimmune skin blistering disorders such as bullous pemphigoid, mucous membrane pemphigoid, and more. The non-inflammatory variant, however, is characterized by minimal inflammation seen clinically or on histology, and presents with blistering, scarring, and skin fragility specifically on trauma-prone areas of the body [17].

## 3. Cutaneous Squamous Cell Carcinoma

### 3.1. Pathophysiology of cSCC

Cutaneous squamous cell carcinoma (cSCC) is the second most common type of skin cancer in the US. The estimated annual incidence of cSCC exceeds one million cases [18] and continues to grow due to increasing risk factor exposure across the nation. Such risk factors include UV radiation and increased prevalence of systemic immunosuppression due to a growing number of immunosuppressive medications or conditions. Additionally, growth in the number of skin cancer screenings performed across the country will also contribute to higher rates of cSCC being detected [19]. cSCC pathophysiology is primarily due to mutations that cause the uncontrolled proliferation of keratinocytes. These mutations usually occur in pathways that regulate cell cycle progression, as well as apoptosis. Most cases of cSCC arise from mutations in the *TP53* gene, which codes for the tumor suppressor protein, p53 [20]. Loss of p53 allows affected cells to circumvent apoptosis, thus resulting in unopposed proliferation of pre-cancerous cells. As a result, p53 mutations are most commonly found in actinic keratoses and cSCC in situ, both of which can mature into cSCC [19]. Regarding metastatic cSCC, the risk in the general population is about 4% compared to those who are immunosuppressed, for whom the risk may be 2 to 3 times greater. Surgical excision remains the most effective treatment modality for cSCC, with recurrence rates below 6% and five-year recurrence-free survival at roughly 90%. Proper excision that is based on clinical margin size remains an important factor in determining success rates. Approximately 95% of tumors less than 2 cm were successfully cured with clinical margins of 4 mm, while cases larger than 2 cm required at least 6 mm margins [21]. Regardless, a properly calculated margin size in surgical excision proves to be one of the most effective curative methods for cSCC.

### 3.2. Pathophysiology of cSCC in EB

Patients with any form of EB are predisposed to skin fragility and blistering that ultimately leads to an endless cycle of chronic wound healing. More specifically, the genetic profiles of different subtypes of EB are primarily responsible for the lack of functional gene products that normally aid in the healing process. Without these proteins, blisters and other wounds that arise from EB are subject to chronic cycles of inflammation and fibrosis that put the skin at risk for the development of cSCCs [11].

In normal skin, wound healing is governed by four primary phases: hemostasis, inflammation, proliferation, and skin remodeling. Hemostasis represents the process by which damage to the skin triggers the formation of a fibrin matrix surrounding the wound area. This is preceded by constriction of the neighboring vasculature to induce platelet aggregation [22].

The formation of platelet aggregates precipitates the release of cytokines to promote leukocyte migration to the wound. This inflammatory response serves as the body’s initial defense against potential microorganisms that have entered the skin via the wound [23]. Macrophages have been shown to play a major role in wound healing and produce additional growth factors and cytokines that result in angiogenic neovascularization, fibroblast proliferation, and migration of epidermal keratinocytes [24].

Within a few days of the injury, the proliferative phase of wound healing begins as fibroblasts enter the wound and produce matrices with high amounts of type III collagen. Furthermore, keratinocytes residing at the basal layer of the wound site secrete proteins that aid in the reconstruction of the basement membrane. These processes are also primarily triggered by the release of various cytokines and other molecular signals necessary for adequate wound healing [11].

Lastly, during the remodeling phase, fibroblasts differentiate into myofibroblasts. These myofibroblasts stimulate contraction of the wound via expression of alpha-smooth muscle actin. The matrix of type 3 collagen that was previously produced during the proliferative phase is now replaced by a more durable and tensile type 1 collagen [22].

The recessively inherited form of DEB has been known to cause more severe phenotypes. According to prior literature, it has been indicated that the skin in RDEB is characterized by an intrinsic pro-inflammatory state [11,25], consisting of generally higher baseline levels of gene products involved in immune system stimulation [11]. A study analyzing monozygotic twins with differing severity of RDEB showed that disease with greater severity, as shown by one twin who had blisters affecting the entire body, may be a result of increased inflammation as compared to a less severe phenotype, as shown by the other twin [26].

In normal skin, senescent cells in the wound site release growth factors that promote the differentiation of fibroblasts into myofibroblasts that ultimately aid in healing. However, in patients with RDEB, repeated blistering and thus wound production and healing render the constant presence of senescent cells harmful to skin repair. These cells have decreased proliferation and lifespan as well as secrete inflammatory signals that ultimately hinder the healing process even further [11]. Moreover, these lesions are frequently colonized by microbiota such as *Staphylococcus* sp, and *Pseudomonas* sp. Immune response to this microbiota can further delay the wound healing process. One study identified the potential for the adaptive T-cell response to aid in clearing infected cells from the wound site. However, in conditions such as RDEB, programmed cell death protein-1 (PD-1)/regulatory T-cell mediated immunosuppression may counteract defense effort and prolong infection clearing, leaving RDEB associated skin wounds more prone to cycles of chronic wound healing [27].

**Figure 1:**
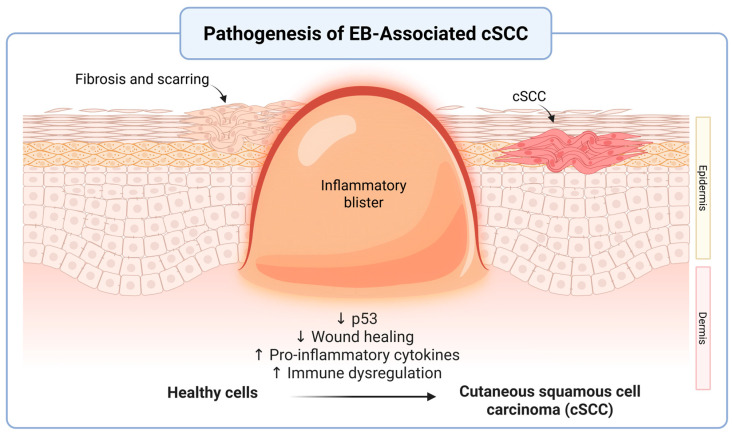
Pathogenesis of Epidermolysis Bullosa (EB)-Associated Cutaneous Squamous Cell Carcinoma (cSCC). Repeated instances of blistering and chronic inflammation in EB support an environment that favors dysregulated tumor growth. These pro-tumorigenic microenvironments are characterized by reduced p53 tumor-suppressor activity, elevated inflammatory markers, immune dysregulation, and decreased overall wound healing. Over time, fibrosis and scarring replace healthy tissue and ultimately contribute to malignant processes such as cSCC. This figure depicts the epidermal-dermal structural changes throughout this process. Created in BioRender. Garrick, J. (2025) https://BioRender.com/s33xcu3 (accessed on 31 July 2025).

While the exact pathophysiology of cSCC in RDEB is unknown, it has been shown that cSCCs in RDEB predominantly arise in areas of excessive skin fibrosis, which is the result of repeated cycles of chronic wound inflammation and repair [10]. Tumor growth factor beta1 (TGF-beta1), the major isoform responsible for the conversion of fibroblasts to myofibroblasts in the wound site, induces the fibrotic environment where cSCCs primarily arise. More specifically, TGF-beta1 that is secreted by dermal fibroblasts have been found to propagate the epithelial-to-mesenchymal transition in RDEB associated cSCC lines, thus contributing to uninhibited tumor growth and metastases [28]. Upregulation of TGF-beta1 also contributes to immunosuppression via regulatory T-cells and inactivation of cytotoxic natural killer cells, allowing tumors to effectively evade immune responses [29]. A study that analyzed the mRNA transcriptome of fibroblasts from RDEB patients found that they housed molecular changes that more closely resemble those associated with neoplastic behavior. Specifically, these cells were able to produce extracellular matrix proteins, cytokines, and other signaling molecules that advance tumor proliferation and progression [30]. Overall, the presence of pro-inflammatory cytokines released by fibroblastic cells uniquely associated with RDEB creates an environment that favors the proliferation of cSCCs that are far more aggressive than their UV light-induced counterparts. A summary of the pathogenesis of cSCC in EB can be visualized in Figure 1.

JEB results from mutations in genes that reduce the production of functioning laminin-332 (LM332) and collagen 17 (COL17). It has been shown that abnormal expression of LM332 has been linked to poor differentiation of cSCC and the creation of an aggressive tumor environment in mouse xenograft models [31]. Only recently has a group been able to demonstrate that mutated LM332 in JEB patients harbors tumorigenic properties and can thus increase the probability of developing cSCCs. More specifically, it was found that adults with JEB have a 1:4 likelihood of developing cSCC beginning from their third decade of life [32]. Furthermore, mutations in COL17, a major component of the dermal-epidermal junction, can result in significant loss of skin integrity, making it susceptible to damage from minor trauma [33]. This can indirectly predispose individuals to develop cSCC due to the role that chronic wound healing has on tumor development [11]. The current literature about the exact mechanism by which JEB induces formation of cSCCs is very scarce. More research is needed to fully uncover these processes and aid in the development of therapeutics for such patients.

### 3.3. Clinical Presentation of cSCC in EB

Amongst the various subtypes of EB, JEB and RDEB are the major variants that result in the formation of cSCCs. While cSCCs in non-EB patients primarily arise from chronic exposure to UV light, cSCCs associated with EB largely originate on sites of prolonged blistering, scarring, and wound formation. Clinically, cSCCs associated with EB are typically more aggressive and, on multiple occasions, have shown increased potential for metastasis. EB-associated cSCCs represent the major cause of death for patients with RDEB, with one Australian EB registry reporting a mere 4-year median survival after the first cSCC diagnosis [10,34].

Because bony prominences are common areas of the body where ulcers and chronic wounds reside, cSCCs have been described to occur on such locations quite frequently. Such locations for primary cSCC lesions include primarily the joints of the hands and feet for both RDEB and JEB [10]. As a result, it can often be difficult to adequately detect early cSCCs in these lesions since their presentation emulates that of any typical ulceration seen in EB. In RDEB specifically, involvement of both upper and lower limbs is the main location for development of cSCC, thus accounting for greater than 90% of all cases [35,36,37]. Since cSCCs associated with EB are generally more aggressive, multifocal lesions are often present at the time of first diagnosis. Moreover, the size of these lesions can also aid in their detection, with cSCC ulceration across all EB variants measuring a diameter greater than 2 cm in 59.1% of cases. Similarly, RDEB lesions can also present with ulcerated lesions larger than 2 cm in 30.4% of cases [38].

Clinical diagnosis of cSCCs in EB patients can be particularly difficult because cancerous lesions can present very similarly to pre-existing ulcerated wounds typical of EB. Often, cSCCs can present within such lesions, making proper diagnosis even more difficult [37].

Since most EB cases are inherited, cSCCs can arise from a very young age, unlike UV-induced cSCCs in non-EB patients [39,40]. Given their higher mortality, early detection of these lesions is integral. The best method for confirmation of any suspicion of cSCC in EB patients is skin biopsy. Preferably, these biopsies should encompass areas surrounding the designated lesion to ensure minimization of sampling error. Histological analysis has demonstrated that, despite the aggressive nature of many EB-associated cSCCs, nearly half of all cases initially present as well-differentiated on pathology but do progress to poorly differentiated later [38].

## 4. Diagnosis of EB-Associated cSCC

The gold standard method of diagnosis of cSCC in patients with or without EB remains skin biopsy. Ideally, punch biopsies that encompass a larger region and preserve the original lesion’s depth are preferred. Moreover, it is important that additional biopsies surrounding the lesion of interest are taken to mitigate sampling error as much as possible. Identification of an cSCC on skin biopsy should be followed by tumor staging and potential regional lymph node biopsy to evaluate metastatic spread given that EB patients are more susceptible to metastases [1,39].

Recent advances in fluorescence diagnosis have shown promise in offering a non-invasive technique for detecting cSCC in EB. Given that cSCC is responsible for early mortality of patients with EB, methods such as fluorescence diagnosis prove crucial in early detection. By using a photosensitizer that localizes specifically to tumorigenic cells, any fluorescence that is captured upon irradiation of specific visible light wavelengths will indicate a neoplastic process. However, one potential drawback of this technology is due to the chronically inflamed state that EB patients harbor. Because the photosensitizer may accumulate in greater amounts in a state of increased inflammation, fluorescence diagnosis may result in false positives regarding the detection of cSCCs [41].

## 5. Management and Treatment

### 5.1. Surgical Excision

Currently, surgical excision of cSCC in EB patients is the most sought-after and definitive treatment form. The exact method of resection is dependent on not only the location of the tumor, but also its stage and aggressiveness. Tumors arising in locations that are cosmetically sensitive or in areas that require the preservation of healthy tissue are often excised via minimally invasive surgical techniques such as Mohs micrographic surgery. Tumors that fail to meet the criteria for Mohs are generally excised via wide local excision [39]. However, tumors that demonstrate involvement of deeper anatomical structures, including vasculature, nerves, or musculature, will likely need more definitive surgical measures, including amputation. In terms of post-surgical wound closure, there have been a variety of different approaches ranging from second intention closure to full thickness grafting, artificial skin equivalents, and more. A case report describing the results between dermal substitute positioning and second intention wound closure demonstrated that dermal substitute had a faster rate of re-epithelialization compared to second intention wound closure [42]. Nevertheless, it is important to consider that wound healing in EB is generally more complicated and thus the appropriate wound closure methods must be chosen accordingly to maximize preservation of quality of life and minimize the risk of postoperative complications.

### 5.2. Immunotherapy

Immunotherapy holds a prominent position in the treatment of metastatic SCC. Because UV-induced SCCs harbor a highly favorable tumor microenvironment that elicits a robust host immune response, immunotherapies such as cemiplimab and pembrolizumab are particularly effective. Cemiplimab, a PD-1 inhibitor approved by the FDA in 2018, demonstrated a 50% and 47% overall response rate for treating cSCC in its Phase I and II trials, respectively. Pembrolizumab, another PD-1 inhibitor recommended by the FDA in 2020, showed a 50% overall response rate when treating locally advanced or recurrent and/or metastatic cSCC [43].

As a result of immunotherapy’s success in UV-driven cSCC, it is also a growing niche of treatment modalities for EB-associated cSCC. A previous case report demonstrated a successful course of treatment of cSCC in a patient with RDEB using cemiplimab. The case presented shows effective control of cSCC progression, with no new lesions having developed while on cemiplimab therapy. Additionally, the patient reported improvement in her chronic RDEB wounds, allowing her to forego opioids for pain management. This case demonstrates how PD-1 inhibitors may play an integral role in the management and treatment of EB-associated cSCC. However, additional widespread studies are needed to more definitively demonstrate its effectiveness [44].

Another study identified clear differences in tumor gene expression and tumor microenvironment in cSCCs from DEB. Compared to cSCCs from immunocompetent patients, DEB-cSCCs displayed significantly lower levels of CD4-positive T cells per mm^2^ while demonstrating increased levels of immune checkpoints proteins and immunosuppressive enzymes such as indoleamine 2,3-dioxygenase (IDO), PD-1, and programmed cell death ligand-1 (PD-L1). These patterns also offer further support for therapeutics such as PD-1 inhibitors as well as additional interventions that can be targeted to specific transcriptomic characteristics of cSCC in DEB [45].

### 5.3. Anti-EGFR Therapy

Epidermal growth factor receptor (EGFR) inhibition is also a growing, albeit less popular, method of treatment of metastatic UV-driven cSCCs. In various clinical trials that investigate the efficacy of EGFR inhibitors, including erlotinib, gefitinib, cetuximab, and panitumumab, panitumumab had the highest objective response rate at 31%, with roughly 13% of patients experiencing a complete response to the treatment [46]. Although these results may not seem as promising as more established immunotherapies, it has been reported that EGFR inhibitors have been better tolerated by patients [47].

Subsequently, EGFR inhibition has also gained traction in the treatment of EB-associated cSCCs. It has been shown that cSCCs in RDEB do indeed express EGFR, albeit at variable levels. Cases of a few patients with cSCCs and lymph node metastases who underwent cetuximab therapy showed improved pain and overall quality of life during the treatment course. The authors propose that early intervention of cSCC treatment in EB patients with cetuximab may prove to be ideal, thus giving patients greater chances of survival [48].

However, given the rarity of EB in general and cSCC in EB, there is a strong lack of larger clinical trials and studies that investigate emerging forms of immunotherapy in these patients. The data regarding these forms of treatment are thus limited to case reports and series. As a result, discussions on the true efficacy of such therapies investigated in large-scale clinical trials will prove useful in determining mainstream methods of treatment, immunotherapy related toxicities, and identifying and addressing challenges in wound healing. The lack of generalizability from smaller scale case reports and studies make efforts to conduct clinical trials essential for ensuring proper guidelines and therapies for this debilitating condition [49].

### 5.4. Electrochemotherapy

Electrochemotherapy is a new method of chemotherapy deliverance that increases the cytotoxic effect of chemotherapeutic agents. By delivering intermittent electrical bursts to tumor tissue, membrane permeability is increased, thus allowing for greater penetration of chemotherapeutic medications [50]. When compared with traditional chemotherapy, electrochemotherapy has the potential to dramatically increase the effectiveness and efficiency with which chemotherapy is administered in patients.

### 5.5. Palliative Care

Because EB does not yet have a cure, palliative care for patients with EB becomes extremely important. Proper wound care, pain and itch management, diet/nutrition are a few of the recommendations that become essential to minimizing discomfort and suffering throughout the course of the disease. Wound care strategies become of utmost importance to prevent infection and further setbacks in wound healing. Because many of these strategies can be similar to those near end-of-life stages, it becomes imperative that proper techniques as outlined by the 2017 Best practice guidelines for skin and wound care in EB are followed throughout the life of the patient [51].

## 6. Surveillance and Prevention

**Figure 2:**
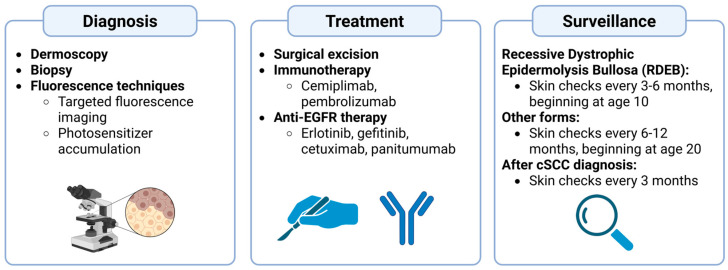
Clinical Pathway for Diagnosis, Treatment, and Surveillance of Epidermolysis Bullosa (EB)-Associated Cutaneous Squamous Cell Carcinoma (cSCC). Currently, the most effective diagnostic strategies include dermoscopy, biopsy, and fluorescence-based techniques to visualize the lesion. Once a malignant lesion has been identified, treatments include surgical excision, immunotherapy, and anti-EGFR therapy. Surveillance before a diagnosis of a malignant lesion may vary by subtype but generally involves skin checks from a young age every 3–12 months, and every 3 months after a diagnosis. Created in BioRender. Garrick, J. (2025) https://BioRender.com/q8ax2nh (accessed on 28 July 2025).

Given the aggressive nature of cSCCs associated with EB, early detection of such lesions is essential. In patients with RDEB, frequent skin checks are recommended once every 3–6 months from age 10. Other forms of EB that are generally less likely to produce severe cSCCs do not need as early and frequent surveillance, and thus skin checks are recommended every 6–12 months beginning at age 20. However, if a malignancy is detected, screenings should occur every 3 months regardless of the patient’s EB status [39]. Moreover, thorough education of skin cancer morphology, especially in EB, and the basics of performing self-skin exams can also be beneficial preventative and early detection measures. A summary of the diagnosis, treatment, and surveillance methods can be visualized in Figure 2.

## 7. Future Directions and Research Needs

Research and development of novel treatment modalities and methods for EB have been a large focus in dermatology research as of late.

Firstly, transcriptomic analysis of RDEB has shown to be promising in identifying potential therapeutic targets. One study identified over 2000 differences in gene expression between RDEB and intact skin, with the former excessive cytokine–cytokine interactions, JAK-STAT receptor pathway activation, and Toll-like receptor signaling. Identifying these targets can then aid in targeting therapeutic interventions to specific deviations that can help restore wounded RDEB skin to normal, intact skin [52]. Given cSCC occurs in RDEB associated wounded skin, this new avenue in treatment and intervention can potentially decrease the prevalence of cSCC in RDEB and streamline the diagnostic process.

Recently, beremagene-geperpavec-svdt (B-VEC) was approved for DEB. B-VEC is a topical, herpes simplex virus type 1 (HSV-1)-based therapy that is designed to deliver the wildtype form of the *COL7A1* gene that is mutated in DEB and thus restore the collagen 7 protein. Results from the trial demonstrated complete wound healing at 6 months of treatment of B-VEC for 67% of patients compared to 22% of placebo controls, and 71% of B-VEC patients and 20% of placebo controls at 3 months of treatment [53]. While it is currently unknown whether the mechanism of B-VEC acts to directly reduce cSCC formation, by dramatically accelerating the wound healing process in DEB, the likelihood of cSCC pathogenesis secondary to DEB will likely decrease as well. As such, treatments of this form may prove beneficial to reducing cSCC incidence in DEB and, thus, mortality in EB patients.

Additionally, there have been studies investigating the use of topical calcipotriol to enhance wound healing in EB [54]. Specifically, the use of topical calcipotriol was demonstrated in a double-blind placebo-controlled trial to increase the endogenous expression of cathelicidin, a chemoattractant for immune cells such as neutrophils [55]. Localizing neutrophil response to areas of chronic wound production and healing is likely to decrease the risk of sustaining bacterial infections in conditions such as EB [56]. Topical diacerein has also been demonstrated to be effective in treating EBS. A randomized placebo-controlled trial showed that only those receiving topical diacerein experienced a greater than 40% reduction in the number of blisters from the initial baseline [57].

Moreover, some treatments for EB that are currently under investigation include ex vivo and in vivo gene therapy, in which the genetic mutations responsible for EB are modified by extracting and reintroducing keratinocytes with the corrected gene, as in ex vivo therapy, or delivery of the corrected gene via a viral vector, as in in vivo therapy. Given the novel nature of these technologies, further studies and research are needed to properly examine the impact that gene therapy can have on EB treatment and reversal [56]. By treating the underlying cause, which is EB, the rates of cSCCs arising directly from the high inflammatory state and repeated cycles of chronic wound damage and healing that characterize this disease can also be decreased, allowing for the prolongation and improved quality of life of the patient.

## 8. Conclusions

Epidermolysis bullosa is a debilitating skin fragility disorder that is usually inherited, although it can, in some cases, be acquired later in life. Characterized by repeated blistering, erosions, and wound healing, these patients are at a significantly higher risk for developing aggressive and oftentimes metastatic cutaneous squamous cell carcinomas. Being able to not only detect and treat these skin cancers early on but also distinguish these lesions from the typical ulcerations and wounds seen in patients with EB becomes critical for their survival. While surgical management is still the gold standard treatment modality, newer advances in research and medicine show promising technologies for treating not just cSCC associated with EB, but EB itself, which can in turn mitigate the mortality caused by cSCCs. Regardless of the variant of EB, a multidisciplinary care approach becomes essential in improving the quality of life of the patient and their families [58]. Proper education about skin cancer prevention, performing self-skin exams, and the clinical morphologies of cSCCs in EB can prove integral to ensuring such lesions are diagnosed and treated as early as possible.

## Abbreviations

The following abbreviations are used in this manuscript:

Epidermolysis bullosa (EB), recessive epidermolysis bullosa (RDEB), junctional epidermolysis bullosa (JEB), epidermolysis bullosa acquisita (EBA), epidermolysis bullosa simplex (EBS), Kindler Syndrome (KS), cutaneous squamous cell carcinoma (cSCC), danger-associated molecular pattern (DAMP), mitogen-activated protein kinase (MAPK).
